# Cancer-Associated Fibroblasts: Epigenetic Regulation and Therapeutic Intervention in Breast Cancer

**DOI:** 10.3390/cancers12102949

**Published:** 2020-10-13

**Authors:** Yeuan Ting Lee, Yi Jer Tan, Marco Falasca, Chern Ein Oon

**Affiliations:** 1 Institute for Research in Molecular Medicine (INFORMM), Universiti Sains Malaysia, Penang 11800, Malaysia; yeuanting@student.usm.my (Y.T.L.); yijer_tan@student.usm.my (Y.J.T.); 2 Metabolic Signalling Group, School of Pharmacy and Biomedical Sciences, Curtin Health Innovation Research Institute, Curtin University, Perth, WA 6102, Australia

**Keywords:** breast cancer, cancer-associated fibroblasts, heterogeneity, epigenetic, post-translational modification, DNA methylation, miRNA dysregulation

## Abstract

**Simple Summary:**

Drug resistance and insensitivity to treatments are the main challenges in breast cancer therapy. Cancer-associated fibroblasts (CAFs) are heterogeneous stromal cells with prevailing roles in cancer development and progression. Epigenetic alterations are essential in regulating CAF activation and heterogeneity. These modifications are druggable targets that can be reversed using pharmacological interventions. CAFs therefore, have a remarkable potential as a therapeutic target in breast cancer. This review provides an update on the mechanisms of epigenetic modulation in breast cancer and discusses the challenges of translating the optimism of CAF-directed therapies from bench to clinic.

**Abstract:**

Breast cancer is the leading cause of cancer-related mortality in women worldwide. Cancer-associated fibroblasts (CAFs) are a heterogeneous population of cells in the solid tumour microenvironment. These cells are positively linked to breast cancer progression. Breast CAFs can be categorised into distinct subtypes according to their roles in breast carcinogenesis. Epigenetic modifications change gene expression patterns as a consequence of altered chromatin configuration and DNA accessibility to transcriptional machinery, without affecting the primary structure of DNA. Epigenetic dysregulation in breast CAFs may enhance breast cancer cell survival and ultimately lead to therapeutic resistance. A growing body of evidence has described epigenetic modulators that target histones, DNA, and miRNA as a promising approach to treat cancer. This review aims to summarise the current findings on the mechanisms involved in the epigenetic regulation in breast CAFs and discusses the potential therapeutic strategies via targeting these factors.

## 1. Introduction

Targeting stromal components in the tumour microenvironment (TME) is the latest hype in the cancer paradigm. The tight interactions between TME and cancer cells form a protective niche that favour cancer formation, progression and dissemination, thereby leading to chemoresistance and therapeutic failures [1].

**Figure 1:**
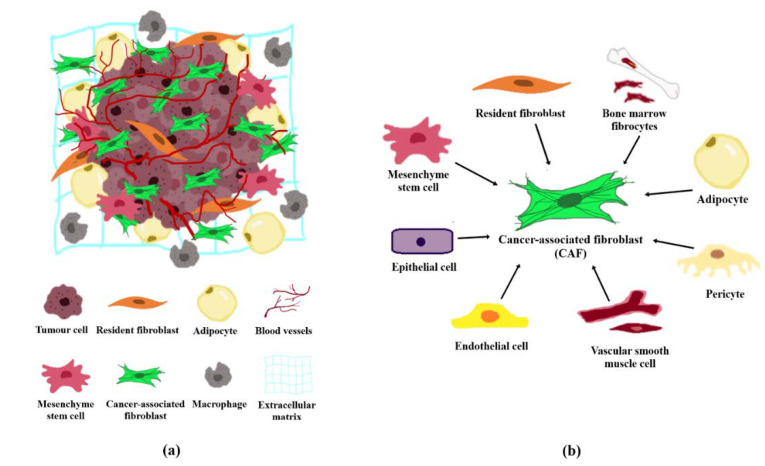
Tumour microenvironment and the origin of CAFs. (**a**) Cellular and non-cellular components in the tumour microenvironment comprising of fibroblasts, adipocytes, macrophages, endothelial cells and the extracellular matrix. (**b**) CAFs may originate from normal resident fibroblasts, mesenchymal stem cells, epithelial cells, endothelial cells, vascular smooth muscle cells, pericytes, adipocytes and/or bone marrow fibrocytes.

The tumour stroma and TME are interchangeable terms that define all non-malignant components in the vicinity of tumours. The tumour stroma consists of cellular components including fibroblasts, adipocytes, lymphoid cells, endothelial cells and mesenchymal stem cells, and non-cellular components such as the extracellular matrix (ECM; Figure 1a). The TME is predominated by cancer-associated fibroblasts (CAFs) which are highly implicated in cancer pathogenesis [2]. CAFs are fibroblast cells within the tumour stroma that exhibit a thin, wavy, and elongated cruciform or stellate-shaped morphology harbouring specific biomarkers [3,4,5]. Compared to normal fibroblasts (NFs), CAFs are larger cells with indented nuclei and possess a more basophilic cytoplasm that contains abundant rough endoplasmic reticulum and free ribosomes, well-developed Golgi complex, and numerous tension fibres [6]. During a histological examination, CAFs may be identified based on its spindle-shaped morphology and the lack of endothelial, epithelial, and leukocyte markers expression, in addition to the absence of specific cancer mutations [7].

CAFs are known to play key roles in cancer progression via secretion of growth factors, cytokines and chemokines into the TME to support cancer cell proliferation and invasion, ECM remodelling and increased angiogenesis [4,8,9]. Traditionally, biomarkers used to identify CAFs include alpha-smooth muscle actin (α-SMA), fibroblast-specific protein 1 (FSP1) also known as S100 calcium-binding protein A4 (S100A4), fibroblast activated protein (FAP), platelet-derived growth factor receptor alpha/beta (PDGFRα/β), tenascin-C (TNC), neuron glial antigen (NG2), desmin, cluster of differentiation 90 (CD90) also known asthymocyte differentiation antigen 1 (THY1), podoplanin (PDPN), discoidin domain-containing receptor (DDR2), and vimentin [4,6,10]. However, these biomarkers are expressed in general populations of fibroblast cells and may not be distinct from CAFs. It was only until recently that asporin, collagen XI-α1 (COL11A1), and microfibrillar-associated protein 5 (MFAP5) were described as markers specific to CAFs [4,11,12,13].

## 2. Breast Cancer and CAFs

According to Global Cancer Statistics (GLOBOCAN), breast cancer (BC) is the most common cancer in women worldwide [14]. BC can be categorised into different subtypes based on the estrogen receptor (ER), progesterone receptor (PR), and human epidermal growth factor receptor (HER2) profiles. Broadly, BC sub-categories include the luminal ER-positive [luminal A (ER^+^, PR^+^, HER2^−^) and luminal B (ER^+^, PR^+^ with nuclear protein Ki-67 expression, HER2^+/−^)], HER2 positive (ER^−^, PR^−^, HER2^+^), and the triple-negative (ER^−^, PR^−^, HER2^−^) [15]. Remarkably, breast cancer-associated fibroblasts (BCAFs) are the most prominent cell type that makes up approximately 70% of the breast tumour volume [16]. Although BCAFs lack the biomarkers that are distinguishable from other CAFs, the presence of specific expression patterns in BCAFs (FAP^+^/α-SMA^+^/CK^−^/CD45^−^) have been frequently observed in the peripheral blood circulating metastatic BC patients [17,18]. Moreover, Ao and colleagues have reported the presence of circulating BCAFs in 30 of 34 (88%) patients with metastasis and 3 of 13 (23%) patients with localised BC, in a pilot study [17]. Emerging studies have since revealed the multiple features and roles of CAFs in various cancers, including BC [2,6,18,19,20,21].

BCAFs have been reported to alleviate BC tumorigenesis through cancer cell reprogramming metabolic regulation, and ECM remodelling. These alterations can eventually lead to enhanced malignant cell proliferation, tumour invasion, and metastasis [18,20,22,23]. According to a study by Mathot and colleagues, the indirect co-culture of various BCAFs (isolated from primary infiltrating ductal or lobular breast carcinoma) with BC cell lines led to the development of aggressive BC phenotypes. These aggressive phenotypes displayed spindle-like morphology with demonstrated reorganised cellular actin [24]. The crosstalk between BC cells and BCAFs encodes a positive feedback loop that facilitates carcinogenesis. BC cells have been reported to activate BCAFs from the surrounding NFs through secretion of platelet-derived growth factor (PDGF) and transforming growth factor-beta (TGF-β). In return, BCAFs may secrete growth factors to fuel tumour growth and confer drug resistance [25].

Compared to mono-cultured BC cells, co-culture of BC cells and BCAFs increased the regulation of FSP1, TGF-β, PDGFβ, fibroblast growth factor 7 (FGF7), interleukins (IL-6 and IL-8), matrix metallopeptidases (MMP2 and MMP11), TIMP metallopeptidase inhibitor 1 (TIMP1), and vascular endothelial growth factor A (VEGFA). These altered regulations subsequently enhanced breast tumour growth, angiogenesis, metastasis, and invasion [26]. The C-X-C motif chemokine ligand 12 (CXCL12) and insulin-like growth factor 1 (IGF1) are CAF-derived cytokines responsible for the pro-survival phosphoinositide 3-kinase-protein kinase B (PI3K-Akt) pathway activation in BC cells. The expression of these cytokines were positively correlated with the predisposition of bone metastasis in triple-negative BCs [27]. Remarkably, BCAFs can also induce endocrine and chemotherapeutic resistance to tamoxifen, doxorubicin, and paclitaxel in BC therapies [18,28].

### 2.1. The Origin of CAFs

The origin of CAFs has been extensively reviewed in recent years, although the mechanism of CAFs activation is still not fully elucidated and remains controversial [1,2,3,19]. In general, CAFs can be derived from precursor cells which include the NFs, mesenchymal stem cells, epithelial or endothelial cells, bone marrow fibrocytes, vascular smooth muscle cells, adipocytes or pericytes [2,7]. The formation of CAFs is attributed to processes involving cancer cells secreted factors, epithelial-mesenchymal transition (EMT) and endothelial to mesenchymal transition (EndMT), and trans-differentiation from other tissues, respectively (Figure 1b).

NFs are the principal source of BCAFs, with approximately 80% of the NFs beingconverted into BCAFs [20,29]. However, the rise of BCAFs can also be attributed to the combined activation of NFs and other precursor cells (as mentioned above) rather than NFs alone [30,31,32,33,34,35]. The formation of BCAFs involves a three-step process, namely (1) the recruitment of distant cells by malignant cells; (2) the reprogramming and conversion of healthy precursor cells into BCAFs through paracrine signalling; and (3) the constitutive maintenance of pro-tumorigenic phenotypes of BCAFs in the TME; which consequently supports tumour progression [36].

### 2.2. BCAFs Heterogeneity

CAFs heterogeneity may be predestined by the CAF precursors cells, [37,38,39,40]. Such heterogeneity may depend on lineage evolution, functional roles, and cancer stages [41]. Growing evidence suggests that different BCAF-subtypes may account for the distinct functional heterogeneity of these cells. BCAF-subtypes that are implicated in tumour proliferation and blood vessel remodelling have been reported to aid tumour metastasis and facilitate apoptosis evasion in genetically modified BC mice models [42]. Interestingly, BC harbouring different mutational status can give rise to BCAF subtypes with diverse functions [43,44]. For instance, BCAFs derived from HER2 positive BC have been found to enhance T47D cell migration (low migratory BC cell line) better, compared to BCAFs isolated from either ER-positive or triple-negative BCs [44].

The CAF-subtypes can be characterised according to their anti-tumorigenic (F1 subtype) or protumorigenic (F2 subtype) activity [45]. However, it remains unclear if the F1 subtype is derived from the resident NFs that resisted conversion into CAFs or if it represents a distinct tumour inhibitory CAF subpopulation, hence warrants further investigation. In a co-culture study between BC and BCAFs, the latter was demonstrated to promote SKBR3, BT20, HCC1937, and MDA-MB-468 BC cell survival, but displayed an opposite effect in MDA-MB-231, MDA-MB-361, Hs578T, and MDA-MB-157 BC cells, suggesting their role as a double-edged sword [46]. Su and colleagues reported a significant reduction of BC cell survival upon co-culture with BCAFs derived from chemo-sensitive tumours (F1 subtype), and vice versa with those isolated from chemo-resistant tumours (F2 subtype), when compared with BC cells monoculture [47]. BCAF subsets with high expressions of GRP77^+^ and CD10^+^ also positively correlated with chemoresistance and poor survival in BC patients [47]. Brechbuhl and colleagues highlighted that BCAFs with modified CD146 protein expression (a stroma cell surface marker) responded differently towards tamoxifen treatments [48]. The elegant study reported the ability of CD146^+^ BCAFs (F1 subtype) to sensitise ER+ BC cells toward tamoxifen, whereas CD146^-^ BCAFs (F2 subtype) enhanced chemoresistance toward tamoxifen by suppressing the estrogen levels in ER+ BCs [48]. Another study highlighted the PDGF-CC paracrine signaling between BC-BCAFs to influence the specification of BC subtypes which affected therapeutic sensitivity [49]. PDGF-CC protein was expressed in abundance in BCAFs and BC cell lines of basal-like subtype but not in the luminal subtype. However, co-culture of the luminal subtype with the BCAFs-conditioned medium revealed a transitional phenotypic switch of the luminal subtype to basal-like subtype BC through the PDGF-CC signaling axis, led to the reduction of the sensitivity to endocrine therapy, supporting the pro-tumorigenic role of CAFs in both subtypes. Collectively, these findings reinforce the functional heterogeneity of BCAFs in exerting either a pro- or anti-tumorigenic phenotype and highlights their indispensable role in influencing the therapeautic outcome in different BC subtypes.

Four BCAF-subsets (CAF-S1-4) have been characterised based on the expression patterns of specific biomarkers [i.e., FAP, α-SMA, FSP1, PDGFRβ, integrin β1 (ITGβ1) and caveolin-1 (CAV1)]. The classification of these BCAF-subsets was also dependent on the BC subtypes (i.e., luminal, HER2 positive, and triple-negative [38]. According to Costa et al., both CAF-S1 and CAF-S4 subsets are prevalent in triple-negative BC. The CAF-S2 subset was enriched in most luminal A tumours, while the CAF-S3 subset was found mainly in breast juxta-tumour. Besides, the CAF-S4 subset was also common in HER2-positive BC cells [38]. The CAF-S1 subset (CD29^Med^ FAP^Hi^ FSP1^Med^ αSMA^Hi^ PDGFRβ^Med-Hi^ CAV1^Low^) expresses all six markers in abundance, except for CAV1. In contrast, the CAF-S2 subset (CD29^Low^ FAP^Neg^ FSP1^Neg-Low^ αSMA^Neg^ PDGFRβ^Neg^ CAV1^Neg^) has low to negative expression of all biomarkers. The CAF-S3 subset (FAP^Neg^ αSMA^Neg^ CD29^Med^ FSP1^Med-Hi^ PDGFRβ^Med^ CAV1^Low^) was only found to have elevated FSP1 expression, while the CAF-S4 subset (CD29^Hi^ FAP^Neg^ FSP1^Low-Med^ αSMA^Hi^ PDGFRβ^Low-Med^ CAV1^Low^) possesses abundant levels of α-SMA and ITGβ1. Nevertheless, the absence of CAV1 expression in all BCAF subsets was found to be positively correlated with the constitutive activation of CAFs phenotype and poor prognosis in BC patients [10,38,50]. Costa and colleagues reported CAF-S1 and CAF-S4 to be enriched in aggressive triple-negative and HER2-positive BCs [38]. The CAF-S1 subset was shown to actively regulate genes associated with cell adhesion, ECM organisation, and immune response which can promote an immunosuppressive TME in BC, whereas the CAF-S4 subset was involved in modulating muscle contraction, oxidative metabolism, and organisation of actin cytoskeleton.

The activation of BCAFs from distinct precursor cells may also lead to different BCAF phenotypes with different functionality. Recently, a total of four BCAF subpopulations were successfully characterised by Bartoschek and colleagues through single-cell RNA sequencing (scRNA-seq) of 768 CAFs transcriptomes isolated from different origins using the mouse mammary tumor virus-polyoma middle tumour-antigen (MMTV-PyMT) BC mice model [51]. The functionality of these BCAF subpopulations was identified by analysing the differentially expressed (SDE) genes using the Gene Ontology (GO) knowledgebase. In this study, BCAFs were classified into vascular CAFs (vCAFs), matrix CAFs (mCAFs), cycling CAFs (cCAFs) and developmental CAFs (dCAFs), based on markers expression, functionality and origin [51]. vCAFs originated from perivascular sources and therefore demonstrated strong expression of genes related to vascular regulation. These include the endothelial PAS domain protein 1 (EPAS1; also known as HIF2A), notch receptor 3 (NOTCH3), collagen XVIII-α1 (COL18A1), and nuclear receptor subfamily 2 group F member 2 (NR2F2) genes. mCAFs were mostly derived from resident NFs demonstratedhigh expression of matrisome genes [i.e., decorin (DCN), lumican (LUM), collagen IV-α1 (COL4A1), fibulin 1 and 2 (FBLN1 and FBLN2), SPARC related modular calcium binding 2 (SMOC2), lysyl oxidase (LOX) and lysyl oxidase-like 1 (LOXL1) genes]. Interestingly, mCAFs also displayed high expression of the CXCL14 gene, which renders their possible involvement in the regulation of tumour-associated immune response. cCAFs have been implicated as the proliferative segments of vCAFs in which only genes involved in the G2, M, and S phase of cell cycle regulation were found to be differentially expressed between the two subpopulation.

Conversely, dCAFs originated from tumour epithelium that has undergone an EMT and therefore highly expressed genes associated with cell differentiation and morphogenesis [i.e., scrapie responsive gene 1 (SCRG1), SRY-box transcription factor 9 and 10 (SOX9 and SOX10)]. Among all, both vCAFs and mCAFs were the most conserved signatures found in most breast cancer patients [51,52]. All four identified CAF subpopulations carried the FAP, FSP1, and α-SMA markers, although dCAFs were the only cell type that did not express the PDGFRβ marker. Valde-Mora et al. have reported the existence of three classes of fibroblasts in the MMTV/PyMT mammary tumour model, based on their functional characteristics. These were the ECM-CAFs with a demonstrated role in tissue remodelling, the inflammatory CAFs (iCAFs) that are involved in immune modulation, and the contractile myofibroblasts which are responsible for cytoskeletal organisation [53]. More recently, two other BCAF populations have been identified based on the selective expression of S100A4 or PDPN markers, termed sCAFs and pCAFs, respectively [54]. Remarkably, the ratio of sCAFs and pCAFs in BC correlated with BRCA mutations in the triple-negative breast cancer and the clinical outcome. Moreover, CAF functionalities are also actively reshaped as tumours advance and spread. At the early stage of tumour initiation in triple-negative 4T1 cancer cell mouse model, sCAFs and pCAFs made up 30% and 70% of total CAFs, respectively. Remarkably, four weeks post-tumour initiation, sCAFs (77%) displayed dominance in composition over pCAFs (23%). The functionality of pCAFs dynamically switched from immunoregulatory to wound healing and ECM remodelling after four weeks of tumour initiation, indicating that CAFs can be educated by the tumour and its microenvironment over time as the tumour evolves. Besides, the PDPN and S100A4 markers co-exist in both estrogen-receptor positive (ER+) BC (luminal subtype) and triple-negative BC (basal-like subtype) patients’ cohort. The low PDPN staining and high S100A4/PDPN ratios were positively associated with BRCA mutation in triple-negative BC but also demonstrated high patient survival rate and better prognosis outcome in both BC subtypes (i.e., ER+ and triple-negative with BRCA mutation BC). Evidently, that pCAFs and sCAFs have respectively distinct pro- and anti-tumorigenic functions in both BC subtypes. In this study, Friedman and colleagues also demonstrated an immunosuppressive role of Ly6C immune-regulatory pCAFs in BC primary tumour while Ly6C- deficient pCAFs had key functions in wound healing [54], suggesting that distinct marker expression within the same BCAF subpopulation may contribute to its functional heterogeneity. The functionalities of different BCAF subpopulations are summarised in Table 1.

## 3. Reprogramming of NFs into CAFs

NFs are quiescent cells involved in the regulation of ECM turnover to facilitate wound healing, tissue inflammation, fibrosis, as well as tumour growth inhibition [6,18]. The acquisition of pro-tumorigenic CAFs phenotypes from NFs is associated with altered expression of a large number of genes from the latter [55,56]. In a comparison study of gene expression between BCAFs and NFs, the MICROMAX^TM^ human cDNA microarray system was selected by Singer and colleagues. They reported BCAFs to exhibit higher expression of genes associated with growth factors and cytokines, intercellular interactions, cell communication and structure maintenance, as well as proteolytic enzyme regulation [57]. In a separate study, Bauer and colleagues used the Affymetrix Human Genome U133 Plus 2.0 and an empirical Bayesian model. Interestingly, twenty-one genes which were closely related to cell paracrine and intracellular signalling, transcription regulation, cell adhesion and migration, were found upregulated in invasive BCAFs isolated from six patients, when compared to NFs [55]. The lack of P53 (TP53), cyclin tumour suppressors including cyclic-dependent kinase inhibitor 1A (CDKN1A, also known as P21), CAV1, as well as phosphatase and tensin homolog (PTEN) protein expression have been reported in BCAFs [20,58]. The compilation of different regulated genes and proteins in activated BCAFs are summarised in Table 2.

Various studies have revealed that disparity in gene expression between CAFs and NFs may be attributed to epigenetic changes [55,60] or genetic alterations [61,62]. However, the mechanisms associated with epigenetic regulations underlying BCAFs have not been extensively studied. Hence, this review aims to summarise past and recent findings on the mechanisms involved in epigenetic changes upon BCAFs transformation. This review will also discuss the potential of utilising epigenetic reprogramming in BCAFs as a novel therapeutic approach in targeting BC.

## 4. Epigenetic Regulation in BCAFs

Epigenetic regulation mechanisms are heritable modifications of chromatin structure or change in gene expression independent of the primary nucleotide sequence [63]. Essentially, epigenetic regulation is vital in sustaining the pre-invasive CAFs phenotype and its heterogeneity. Recent studies have shown that the transition of NFs to activated fibroblasts may be regulated epigenetically through a ‘reversible’ or ‘irreversible’ activation process [5]. Fibroblasts that are reversibly activated are known as the normal activated fibroblasts (NAFs). NAFs have high expression of α-SMA and vimentin proteins which corroborate with its function to promote wound healing in healthy tissues. On the other hand, CAFs are the products of irreversible activation in fibroblasts. The epigenetic-driven hyperactivation of fibroblasts often acquires specialised ECM remodelling capability, robust autocrine activation, dynamic immunomodulatory signalling functions, enhanced anti-apoptotic pathways, and proliferative properties. The constitutive activation of this supposed repair pathway eventually leads to cancer fibrosis. However, the comprehensive mechanism governing the activation of CAFs via epigenetics remains elusive. Yet, studies have shown that CAFs could be epigenetically regulated through TME-driven pathways, cancer cells-CAFs crosstalk and CAFs metabolic reprogramming [64]. The net effects of the paracrine signalling involving cytokines, exosomes, and metabolites between the cancer cell-CAFs interface also provide a platform for CAFs activation [65]. Some of the vital CAF-activating factors released by cancer cells are TGF-β, PDGF, IL-6, IL-1β, epidermal growth factor (EGF), and lysophosphatidic acid (LPA) [10,66].

The bidirectional crosstalk between tumour cells and CAFs may be facilitated by tumour-derived exosomes (TDEs) [37,40,67]. TDEs are extracellular vesicles that are secreted by the cancer cells or the stroma to transport various bioactive molecules including DNA, microRNAs, proteins, lipids, and metabolites, to facilitate intercellular communication between neighbouring or distant cells [68,69]. The exosomes released from CAFs can be internalised by the cancer cells to supporttumour progression, whereas the exosomes secreted by the cancer cells could stimulate CAF activation [67]. TDEs participate in TME remodelling, angiogenesis, invasion, metastasis, and drug resistance [70,71]. In BC, TDEs are utilised by BCAFs to transport miRNA, mRNA, and proteins to BC cells for cancer progression [71,72]. Intriguingly, TDEs are also described as vehicles that transport miRNA-9 (miR-9) from BC cells to breast NFs during BCAF activation in MDA-MD-231 (triple-negative BC) cells [72]. The pro-metastatic miRNA-9 could stimulate the transformation of NFs to BCAFs to ultimatelypromote an aggressive BC phenotype [72]. Furthermore, the ability of TDEs to transport both TGF-β and IL-6 oncogenic factors may lead to BCAFs transdifferentiation [10,73].

Alterations within the TME, such as the elevated amount of reactive oxygen species (ROS) or hypoxia, can result in CAFs activation via metabolic reprogramming. The presence of a reactive TME may elevate both hypoxia-inducible factor 1 alpha (HIF-1α) transcription factor and CXCL12 chemokine level; while decreasing CAV1 expression in CAFs [10,74]. The eventual loss of CAV1 expression in activated CAFs could stimulate TGF-β production and trigger constitutive activation of CAFs [74]. Besides, the development of hypoxic CAFs have been linked to enhanced migration and invasion capabilities [75]. Hypoxia-induced BCAFs were revealed to possess a high expression of G-protein estrogen receptor (GPR30), which can further support proliferation, metastasis, invasion, and chemoresistance in BC cells [76,77].

**Figure 2:**
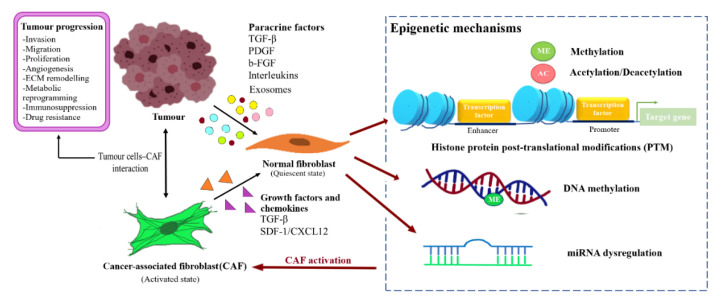
Reprogramming of NFs into CAFs through epigenetic regulation. Fibroblast activating factor secreted by the tumour cells or CAFs caninduced transformation of the normal resident fibroblast into CAFs through epigenetic modulation, and subsequently support tumour progression.

BCAFs can be activated through altered expression of imperative genes and influenced by factors such as the profile of TME and TDEs. The mechanisms underlying epigenetic regulation in BCAFs activation are the post-translational modifications of histone proteins, methylation of DNA, and miRNA regulation. The factors affecting NFs reprogramming and CAFs activation are illustrated in Figure 2.

In general, CAFs do not possess genomic alterations but the presence of epigenetic dysregulations including local DNA hypermethylation and global DNA hypomethylation can change the CAFs transcriptional activity, leading to the acquisition of pro-tumorigenic phenotype [78]. Secreted factors from either the cancer cells or CAFs can serve as a bridge between BC and CAFs to induce gene or epigenetic modification in both cell types [24]. Yet, the mechanism underlying the epigenetic dysregulation as a result of the crosstalk in cancer remains unknown. The CAF-secreted factors are capable of activating hypermethylation at the cpG island downstream of the transcription start site (TSS) related in BC delineating the significance of epigenetic marks in CAF-dependent BC reprogramming [24].

In-direct co-culture using BCAFs conditioned media on MCF-7 and MDA-MB-231 cell lines resulted in increased expression of TGF-β1, which promoted EMT by up-regulating the expression of IncRNA HOX transcript antisense RNA (HOTAIR) expression in BC. The HOTAIR then mediated H327K tri-methylation of CDK5RAPI and EGR-1 promoters region leading to the elevation of CDK5 protein expression to promote BC invasion and migration [79]. Mounting evidence has revealed the importance of TGF-β serves as major conduit for epigenetic change through BC-BCAFs crosstalk [24,56,79,80,81,82]. The dynamic interactions of histone acetyltransferase (HAT), histone deacetylases (HDACs), histone demethylases (HDMs), histone methyltransferase (HMT) with regulatory-SMAD (R-SMAD) effectors in response to TGF-β signalling are able to modify the gene expression patterns [80]. The interaction of P300/CBP and R-SMAD then leads to the acetylation of SMAD protein, to upregulate the genes downstream of the TGF-β signaling axis. On the other hand, the interaction of R-SMAD with HDAC could suppress the TGF-β responsive genes [80]. The TGF-β-regulated genes have been associated with various aspects of tumorigenesis including cell proliferation, inflammation, EMT, metastasis and invasion [83,84,85].

### 4.1. Post-Translational Modification of Histones in BCAFs

Histones can undergo post-translational modifications (PTM) to alter gene expression and cell behaviours. Generally, PTM comprises of methylation, phosphorylation, acetylation, ubiquitination, and sumoylation [86]. Epigenetic enzymes that are responsible for the addition of epigenetic marks are referred to as “writers”, such as HATs and HMTs that catalyse acetylation and methylation respectively. On the contrary, HDACs and HDMs are accountable for the removal of acetyl and methyl groups, respectively and are known as “erasers” [82]. The acetylation of histone H3K9, H3K36, H3K27, H3K18, and H4K12 are often associated with gene activation; although deacetylation by HDACs can also turn off the gene transcription processes. Conversely, the demethylation of histones can either stimulate (e.g., H3K4me3) or repress (e.g., H3K9me3, H3K27me3) gene transcription [86,87].

In BC, histone modification through hypomethylation can lead to BCAF activation. Ina co-culture study, MDA-MB-468 BC cells triggered NFs to secrete A disintegrin and metalloproteinase with thrombospondin motifs 1 (ADAMTS1) enzyme which promoted cancer invasion [88]. The increased ADAMTS1 expression was linked to HMT deregulation, which resulted in vasionin NAF200N NF cells in H3K27me3 trimethylation and subsequent reduction of transcriptional suppressor known as the Enhancer of zeste homolog 2 (EZH2). The reduced EZH2 levels lead to the upregulation of the expression of ADAMTS1 dependent genes and proteins, which could positively impact tumour growth, cell migration, invasion, and transformation of NFs to CAFs [88].

### 4.2. DNA Methylation in BCAFs

DNA methylation is a covalent modification of the DNA molecule through the addition of methyl groups at the 5’cytosine-phosphate-guanine-3’ (CpG) regions by DNA methyltransferases (DNMTs), usually for transcriptional repression [86]. The three types of DNMTs responsible for DNA methylation are DNMT1, DNMT3A, and DNMT3B [89]. Although DNA methylation lowers the binding affinity for transcription factors, the predominant transcription suppression is mediated by the methyl-DNA-binding proteins (MBPs). In general, MBPs recognise and bind methylated DNA to initiate transcription silencing through the recruitment of other repression factors, including HDACs [90,91]. The three major families of MBPs are the methyl-CpG-binding domain (MBD) proteins, SET, and RING finger-associated (SRA) domain proteins, and methyl-CpG-binding zinc finger (ZnF) proteins [90]. DNA methylation is also divided into two classes: DNA hypermethylation (gene silencing) and DNA hypomethylation (gene activation) [92].

DNA methylation in chromatin regulation can affects BCAF gene expression and subsequently cell behaviour. For instance, the methylation of progesterone receptor (PGR), hydroxysteroid 17-beta dehydrogenase 4 (HSD17B4), and cadherin 13 (CDH13) genes in both HER2 positive BCAFs and tumour cells was found to enhanced BC progression and invasion [93]. In addition, Hu and colleagues discovered distinct DNA hypomethylated genes in BCAFs, NFs, and breast tumour epithelial cells [60]. Using the methylation-specific digital karyotyping (MSDK) method, they found the transcriptional regulator PR/SET domain 14 (PRDM14) and transcription factor homeobox D4 (HOXD4) genes to be exclusively methylated in tumour epithelial cells and NFs respectively. They also found methylated hypothetical protein LOC389333 gene in both the tumour epithelial and NF cells, as well as hypermethylated transmembrane protein 187 (TMEM187 or CXorf12) gene in the tumour stroma cells. Interestingly, both solute carrier family 9 isoform A3 regulator factor 1 (SLC9A3R1) and CDC42 effector protein 5 (CDC42EP5) genes were methylated more frequently in the stromal fibroblasts compared withbreast tumour epithelial cells [60]. SLC9A3R1 is a tumour suppressor protein that has been reported to be disrupted in approximately 58% of BC cell lines [94]. The translated product of the CDC42EP5 gene also plays a role in septin regulation which is vital for CAF activation [95,96]. However, the role of CXorf12 protein in BCAFs remains undetermined to this day.

Based on the investigation by Onuchic and co-workers, active DNA methylation in BCAFs was found to stimulate collagen I-α1 (COL1A1), fibronectin 1 (FN1), and FAP gene expressions [97]. The upregulation of these genes is linked to ECM progression and metastasis [6,98]. In another context, DNMTs may act as regulators for the pro-invasive activity of BCAFs. Albrengues and colleagues demonstrated that BCAFs pro-invasive activity was sustained by the constitutive activation of Janus kinase 1/signal transducer and activator of transcription 3 (JAK1/STAT3) signalling pathway, as a result of STAT3 and DNMT3B activation. Furthermore, three tumour suppressor genes: TGF-β1 receptor type 2 (TGFBR2), THY1 and PTEN genes have been shown to be hypomethylated and deactivated in BCAFs [9]. Taken together, these findings reinforced the role of epigenetic regulation in conferring a pro-tumorigenic BCAF phenotype.

### 4.3. miRNA Regulation in BCAFs

miRNA regulation is the most extensively studied and well documented epigenetic mechanism in BCAFs. miRNAs are short (17–24 nucleotides) noncoding regulatory RNAs that are involved in the regulation of post-transcriptional target gene expression [99,100]. Briefly, miRNA binds the 3’ region of mRNA to inhibit protein translation or induce degradation of the former. Mounting evidence has revealed the involvement of dysregulated miRNAs in the promotion of CAFs phenotype in various cancers including oesophageal squamous cell carcinoma (SCC), ovarian, gastric, lung, prostate, endometrial and BC [9,101,102,103,104,105,106].

In BC, miRNA dysregulation increases cancer cell motility and reprogramming of NFs to BCAFs. The suppression of the miR-200 family in BCAFs has been shown to increase cancer cell migration and invasion, due to their compromised roles in modulating ECM remodelling, EMT regulation, and cell migration [99,106]. The dysregulation of miR-200 family members miR-200a, miR-200b, miR-200c, and miR-141 has also been reported to initiate BCAFs phenotype acquisition via increasing α-SMA and FAP expression [106]. Additionally, the conversion of NFs to BCAFs involves the reduction of mediator miR-146p-5p and the cyclin-dependent kinase inhibitor 2A (CDKN2A or P16INK4A) gene, which results in a surge of IL-6 level [107].

The dysregulation of miR-31 has also been reported to positively correlated to the overexpressed special AT-rich sequence-binding protein 2 (SATB2) gene in BCAFs [39,101]. The upregulated SATB2 genes were used as a determinant to represent activation of genes involved in cell migration and invasion, and reprogramming of NFs into BCAFs [101]. On the contrary, miR-9 was associated with cell motility, ECM remodelling, and the migration and invasion capabilities of NFs. The inhibition of miR-9 leads to the impairment of these capabilities in BCAFs [72]. The dysregulation of miRNA in BCAFs can also interfere with the production of paracrine signalling factors such as TGF-β1 and IL-6 to influence cancer progression, metastasis, and drug resistance [4] (Table 3).

## 5. Therapeutic Intervention Targeting Epigenetics in BCAFs

The current emphasis on discovering therapeutic methods that target BCAFs by interfering in their epigenetic mechanisms could be an alternative strategy for BC therapy. One emerging interest is to develop new agents that can reversibly target epigenetic inheritance, either as standalone drugs or as adjuvants for cancer therapy [115,116,117]. The plasticity of CAFs could be exploited to shift the tumour-promoting subtype to a dormant state or acquire tumour suppressive phenotype, by targeting specific signalling pathways and tumour-derived factors [1,118]. Reprogramming of BCAFs back to NFs could be a potential treatment strategy since epigenetic modifications are malleable, plastic, and potentially reversible. For example, activated CAFs treated with all-trans retinoic acid (ATRA), calcipotriol, and minnelide displayed restoration to a quiescent state, resulting in improved therapeutic outcome in PDAC [41,119,120,121]. Impediment of both DNMT activities and JAK signalling in vitro and in vivo was also revealed to reprogramme invasive BCAFs back to an inactive state [9]. Therefore, therapeutics directed at epigenetic regulations are appealing as this process is reversible, in contrast to targeting treatments at DNA mutations. The restoration of epigenetically dysregulated genes may be beneficial to repress cancer or sensitise cancer towards therapies [64].

Epigenetic drugs such as DNMT inhibitors (DNMTi) and HDAC inhibitors (HDACi) are currently undergoing clinical trials and have demonstrated abilities to reactivate epigenetically-inactivated genes [122]. DNMTis are cytidine analogues that block DNA methylation and degradation through the obstruction of DNMT activities [123]. Similarly, HDACis inhibit HDACs and hence are capable of increasing the global histone acetylation patterns [64]. Both DNMTis and HDACis, therefore, display significant effects on cancer cell apoptosis, cell cycle arrest, and tumour progression [124]. To date, two DNMTis [Azacitidine (Vidaza) and 5-Aza-2’-deoxycytidine (Dacogen)] and five HDACis [Vorinostat (Zolinza), Romidepsin (Istodax), Belinostat (Beliodaq), Panobinostat (Farydak) and Chidamide (Epidaza)] have been approved by the Food and Drug Administration (FDA) for the treatment of myelodysplastic syndromes and T-cell lymphomas [124,125].

DNMTi may be exploited to target CAFs with active hypermethylation or hypomethylation. However, the treatment of CAFs with DNMTi alone may not be as effective. Numerous studies have shown that the Dacogen (DNMTi)-treated CAFs exhibit lower DNMT levels, but do not display total gene reactivation associated with inhibited promoter methylation [126,127,128]. The lack of responsiveness towards Decogen could be due to the plasticity and undifferentiated properties of CAFs, which result in reduced DNA methylation compared to cancer cells [126]. Conversely, Vidaza (DNMTi) has been reported to be a carcinogen which promotes tumorigenesis in vivo [129]. Vidaza wasalso capable of enhancing the invasiveness and induced pro-metastatic gene expression in non-invasive MCF7 and ZR-75-1 BC cell lines in vitro [130]. The exclusive depletion of DNMT1 induced p53-dependent apoptosis in fibroblasts while reduced BC cell survival in vitro could provide inference for novel DNMTi design and the proper utilisation of current inhibitors [130,131].

Similarly, the use of HDACi such as vorinostat on CAFswas found to activate pro-tumorigenic factors [132,133,134]. CAFs treated with HDACi alone showed increased secretion of pro-inflammatory tumour supportive cytokines and chemokines [134]. High concentrations of Vorinostat (HDACi) also triggered NF-κB signalling mediated senescence-associated secretory phenotype (SASP) in BCAFs, which led to enhanced tumour growth in vivo [133]. On the other hand, BCAFs exposed to Scriptaid (HDACs 1/3/8 inhibitor) possessed lower CAF marker expression, reduced ECM secretion, and impaired co-cultured BC cell invasion in vitro [135]. A study by Li and colleagues also linked HDAC6 upregulation in BCAF to support an immunosuppressive TME; in which HDAC6 inhibition effectively restored the anti-tumour immunity of the TME toinhibit BC tumour growth [136]. Nevertheless, complications arising from the ineffectiveness of DNMTi and undesirable tumorigenic effects by HDACi in sole treatments may be resolved using different agents in combination. Yu and colleagues demonstrated a more effective reactivation of methylation-repressed genes in CAFs treated with DNMTi and HDACi in combination compared to DNMTi alone [126]. Nyugen and collaborators also highlighted the effectiveness of utilising combined treatment of HDACi and other signalling pathway inhibitors to attenuate the inflammatory phenotypes in CAFs and to further improve treatment efficacy [134]. Other epigenetic drugs targeting the “writers” such as the EZH2 inhibitors (GSK2816126 and CPI-1205) and the “erasers” including histone lysine demethylase inhibitors (GSK2879552 and pargyline) are still under development or are currently in clinical trial [86,137,138]. Both tazemetostat (EZH2 inhibitor) and gefitinib (a histone lysine demethylases inhibitor) have been recently approved by the FDA for the treatment of epithelioid sarcoma and non-small cell lung cancer (NSCLC) respectively. However to date, no study has been performed on the use of these drugs against BCAFs in vitro or in vivo. Both DNMTis and HDACis are being tested on BC in phase I and II clinical trials either as monotherapy or in combination [116,139,140,141,142,143]. Although monotherapy with epigenetic drugs displayed limited efficacy in BC patients, reduced tumour DNA methylation, elevated histone acetylation, and gene reactivations have been observed [116]. Therefore, further investigations on the use of epigenetic drugs on BCAFs are essential.

The implications of miRNA and TDE on cancer progression are not unfamiliar to us. Recent discoveries of tumour suppressive small interfering miRNAs (such as mimics of miR-16, miR-29, and miR-34) and antisense oligonucleotides (such as anti-miRNA 103/107, 122 and 155) have enabled the development of novel anti-cancer drugs, which most are still undergoing clinical trials [144]. The use of miRNA mimics and antisense oligonucleotides against dysregulated miRNAs in BCAFs have shown promising results to be developed as BC therapeutics. A recent study by Santolla and colleagues demonstrated successful inhibition of miR-221-induced cell growth and migration in clinically derived BCAFs upon (LNA)-i-miR-211 oligonucleotide treatment [112]. Al-Harbi and co-workers also reported the restoration of anti-tumour miRNA let-7b (which is deregulated in CAFs) by let-7b mimic in BCAFs to culminate in the inhibition of paracrine pro-tumour effects [145]. Although BCAFs exosomal miRNA can increase stemness and EMT phenotype in BC cells, these effects were reverted in BT549, MDA-MB-231, and T47D BC cell lines after treatment of clinically derived BCAFs with anti-miR21, -143 and -378e in vitro [71]. The downregulation of miR-146b-5p was also linked to the formation of BCAFs and was responsible for the paracrine pro-invasive/migratory effects of BC cells. However, therestoration of miR-146b-5p using curcumin on patient-derived BCAFs (CAF-87) impeded CAFs markers and their paracrine pro-tumour potential [146,147]. The inhibition of miR-9 in primary triple-negative BCAFs with LNA-9 also hindered its migration and invasion capacity. Despite the advances in research that highlight the potential of targeting miRNA in BC therapeutics, many RNA-targeted therapies are not able to reach clinical trials, and none have been approved by the FDA [148]. The complexity of miRNA regulation and its function in different CAFs may further complicate the clinical development of RNA-targeting agents [149], which warrants further investigation.

Another therapeutic strategy would be targeting the paracrine signalling factors such as the CAFs-secreted TGF-β and CXCL12, and cancer cells derived PDGF-α/β, b-FGF, and IL-6 that could lead to CAFs activation [20,86,150]. In the case of BC, factors including EGF, FGF2, and CXCL ligands are involved in the epigenetic regulation of BCAFs activation and hence are noteworthy therapeutic targets. One crucial molecular pathway that was reported to influence histone modification and DNA methylation in BCAFs was the TGF-β signalling pathway. TGF-β signalling modulated epigenetic alterations through the SMAD family of proteins as well as epigenetic regulators, including the c-SKI and SnoN proteins [56,82]. In fact, the co-inhibition of HDACs and TGF-β signalling pathway activator protein 1 (AP-1) expression was reported to improve the efficacy of HDACi *in vitro* [134]. Currently available TGF-β inhibitors targeting BCAFs include resolimumab, galunisertib and tasisulam, which are still under clinical trials [151,152]. In addition, the CAF phenotypes and functionalities may also be influenced by TDEs released by the tumour cells at different stages of development, to fuel tumour progression [153]. In CRC, the CAFs activated by late-stage exosome (SW620-Exos) exhibited a more aggressive and invasiveness characteristic. In contrast, CAFs activated by early-stage exosome (SW480-Exo) demonstrated pro-proliferative and pro-angiogenesis abilities [153]. Therefore, targeting tumour-stage specific TDE could be utilised in designing BCAF-directed therapy to prevent the reprogramming of NFs into cancer-promoting cells.

The recent emergence of the tumour-suppressing CAF phenotype (F1 subtype) has redefined the conventional view of CAFs as pro-tumour entities. While the tumour-promoting (F2) subtype has long been taking centre stage, the F1 subtype has lately garnered the interest of scientists as a therapeutic target. Rhim and colleagues have demonstrated the tumour-suppressive role of the Sonic hedgehog (Shh)-expressing stromal components in impeding tumour growth via restricting angiogenesis, at least in PDAC [154]. The tumour-suppressive role of CAFs has also been exhibitedin cancers of the prostate, lung, and melanoma [155,156]. Keeping in view that CAF subtypes may have overlapping gene signatures, caution should be practised especially when selectively targeting CAF subtypes expressing distinctive markers particular to pro-tumorigenic phenotypes, such as GD10^+^, GPR77^+^, FAP^+^, and S100A4^+^ [38,42,47]. The expression of Procollagen-Lysine, 2-Oxoglutarate 5-Dioxygenase 2 (PLOD2), and Rho GTPase-activating protein 26 (ARHGAP26) could be employed as the specific CAF signatures in non-inhibitory and inhibitory fibroblasts, respectively [157]. Nonetheless, the lack of specific markers to distinguish tumour-promoting and tumour suppressing CAF phenotypes continues to be a hurdle in designing therapies that can distinguish between the two CAF subtypes accurately. To overcome this challenge, the employment of the CAF subtype-specific lineage tracing model may assist in revealing the origin of distinct CAF populations [41].

CAF subtypes possessing similar genetic landscapes, but distinct expression profiles may lead to diverse functionalities, which may influence therapeutic response [38]. Thus, combination treatments targeting co-existing subpopulations based on selectively expressed cell markers can be deployed to circumvent drug resistance to achieve therapeutic success. Similar CAF subtypes in different tissue types may play different roles attributable to different CAF marker expression levels between tissues, which further increased the complexity and heterogeneity of CAF populations [158]. Twelve CAF markers ((DLG1, BHLHE40, ROCK2, RAB31, AZI2, PKM2, ARHGAP31, ARHGAP26, ITCH, EGLN1, RNF19A, and PLOD2) have been discovered to overlap across five different cancers, (i.e., breast, colorectal, lung, basal cell, and squamous cell carcinoma) [157]. Identification of these marker expression patterns on CAFs could shed light on the functional roles of these CAF subtypes in cancer progression, thus opening a new avenue for the development of treatment strategies.

## 6. Conclusions and Future Perspectives

BCAFs play prevailing roles in cancer progression and are imperative components in the TME, especially in their aptitude to complicate clinical prognosis and treatment response. Considering the implication of BCAFs in BC progression, they, therefore have tremendous potential as a therapeutic target in breast cancer. The ability to discriminate the pro-tumorigenic (F2 subtype) BCAFs from the anti-tumourigenic (F1) subtype is critical, in order to apply the selective inhibition concept by eliminating the cancer-promoting subtype in favour of the cancer-suppression subtype. To date, the distinct BCAF markers associated with F1 and F2 subtypes phenotypes remain unclear. The biological events and mechanisms involved in the programming of the tumour-suppressing subtype-1 are also not well understood. Both BCAF-subtypes may share similar expression of CAF-derived factors or transcriptional signatures, but these molecules may act in opposite manners in different BC, owing to the complexity of the CAF intrinsic properties, TME, and malignant tissue type. In addition to the wealth of knowledge on reported BCAF subpopulations, many candidates remain undiscovered. This opens an avenue for new discovery and investigation into the relationship between various subpopulations and their roles in cancer. The choice of suitable models and techniques that take into consideration the crosstalk within the tumour microenvironment is critical to facilitate the development of CAF-directed therapies [7,41]. The fundamentals of CAFs recruitment and the dynamics of CAFs-stromal interaction could also be unmasked using the allele and intravital imaging techniques [118].

Albeit the variance in epigenetic regulations that give rise to heterogeneity in BCAFs, the underlying mechanisms governing BCAFs have been gradually revealed. However, the mechanisms underlying the epigenetic regulation of unique gene expression patterns and biological behaviour of CAFs remains unclear. The BCAFs heterogeneity and subtypes can exhibit differential expression of genes that affect tumour aggressiveness and immunosuppressive ability, thus investigating their lineages could provide answers to their origins and help in the design of stromal therapeutics. In addition, the distinctive clusters from the heterogeneous BCAF subtypes may also confer contradicting effects (pro- or anti-tumour) toward BC tumour in the TME, causing therapeutic failure. The involvement of BCAF subtypes and the scarcity of unique biomarkers to identify these subpopulations adds a further degree of complexity. Moreover, the lack of distinct yet specific BCAF markers that are exclusively different from other tumour stromal cells also poses a great challenge in targeting BCAFs as a therapeutic modality. The co-existence of similar CAF populations across other cancers sharing similar markers such as S100A4^+^ and PDPN in breast and CRC cancer [54,159], suggests that CAFs may possess overlapping functions in different cancers thereby affirming the necessity to redefine CAF classifications using an improved nomenclature system which takes into account specific marker expressions in CAF subpopulations and their functionalities [7]. The single-cell RNA-sequencing (scRNA-seq) and DroNc-seq technique can also be employed to investigate markers that are distinctive to CAF subtypes to address the problem of CAF heterogeneity [160]. Nonetheless, targeting several marker combinations instead of a single specific marker on BCAFs could be incorporated in the design of fibroblast-directed therapies to improve treatment efficacy.

Finally, the lack of FDA-approved epigenetic drugs also warrants for concerted efforts between universities and industrial partners to develop new drug candidates for the treatment of solid tumours. The advancement of technologies such as Methylation-Specific PCR (MSP), Combined Bisulfite Restriction Analysis (COBRA), Methylation-sensitive Single Nucleotide Primer Extension (MS-SNuPE) ChIP-seq, and Formaldehyde-assisted Isolation of Regulatory Elements (FAIRE) have also allowed more accurate genome-wide assessment of epigenetic modifications. These high-throughput datasets are beneficial for information-mining towards the identification of more specific biomarkers. On the whole, the issues mentioned above are the critical challenges associated with CAFs biology and epigenetics that deserve further investigation to facilitate the translation of CAF-directed therapy from bench to clinic.

## Figures and Tables

**Table 1:** List of BCAF subpopulations in breast cancer.

Subpopulation (s)	Functionality	Marker (s)	Ref.
CAF-S1	Immunosuppression	FAP, αSMA, CXCL12, 1L6	[38,41]
CAF-S2	N/A	Low to negative expression of all biomarkers	
CAF-S3	N/A	PDGFRβ	
CAF-S4	Contractile signature, oxidative metabolism	αSMA, CD29	
vCAFs	Vascular development and angiogenesis	NID2, CD31, αSMA, PDGFRβ	[41,51]
mCAFs	Provider of extracellular matrix and EMT	PDGFRα, FBLN1	
cCAFs	Engage in cell cycle and cell division, angiogenesis	Ki-67, PDGFRβ	
dCAFs	Cell differentiation and development	SCRG1, SOX9	
ECM-CAFs	EMT, ECM interaction and tissue remodelling	TNC	[41,53]
Inflammatory CAFs	Immunomodulation, monocyte recruitment	Ly6C, C3, CXCL12, PDGFRα	
Contractile myofibroblasts	Actin cytoskeleton organisation	αSMA, MYLK	
sCAFs	protein-folding and metabolic regulation	S100A4	[54]
pCAFs	immune regulation, cell migration, wound healing	PDPN	

Abbreviations: BCAF, breast cancer-associated fibroblast; FAP, fibroblast activation protein; αSMA, alpha smooth muscle actin; CXCL12, C-X-C motif chemokine 12; 1L6, interleukin-6; PDGFRβ, platelet-derived growth factor receptor beta; CD29, cluster of differentiation 29; vCAFs, vascular cancer-associated fibroblasts; mCAFs, matrix cancer-associated fibroblasts; cCAFs, cycling cancer-associated fibroblasts; dCAFs, developmental cancer-associated fibroblasts; NID2, nidogen-2; CD31, cluster of differentiation 31; PDGFRα, platelet-derived growth factor receptor alpha; FBLN1, fibulin-1; Ki-67, antigen Ki-67; SCRG1, scrapie responsive gene 1, SOX9, SRY-Box transcription factor 9; TNC, tenascinC; Ly6C, lymphocyte antigen 6C1; C3, complement component 3; MYLK; myosin light chain kinase; S100A4, S100 calcium binding protein A4; PDPN, podoplanin; N/A, not applicable.

**Table 2:** Alteration of gene and protein expressions upon acquisition of BCAF phenotype from breast NFs.

Protein/Genes	Regulation	Ref.
**Growth Factors and Cytokines**	Up	[57]
Granulocyte-macrophage colony-stimulating factor receptor (GMCSFR), Pigment epithelium-differentiation factor (PEDF), Kirsten rat sarcoma viral oncogene homolog protein (K-RAS), Effector cell protease receptor 1 (EPR-1), Hepatoma transmembrane kinase ligand (HTK ligand), Alpha-2-macroglobulin receptor-associated protein (RAP)		
**Cell-Cell Interaction, Communication and Cell Structure Maintenance**	Up	
Osteopontin (OPN), Transmembrane 4 superfamily protein (SAS), Tapasin (NGS-17), Troponin I fast-twitch isoform (TNNI2)		
**Proteolytic Enzymes Regulation**	Up	
Dihydrodiol dehydrogenase (DHDH), Protein tyrosine phosphatase (PTP), Hematopoietic consensus tyrosine-lacking kinase (HYL) Pro-cathepsin L (zymogen of proteolytic enzyme cathepsin L), Beta-D-galactosidase (β-gal), Tryptophanyl tRNA synthetase (IFNWRS), Rolipram-sensitive 30, 50-cyclic AMP phosphodiesterase		
**Cell Cycle and Metabolism**	Up	
Guanine nucleotide-binding protein G(q) subunit alpha (GNAQ), Circadian locomoter output cycles protein kaput (CLOCK), 63-kDa protein kinase related to rat ERK3; also known as mitogen-activated protein kinase 4 (MAPK4), Phospholipase A2 (RASF-A PLA2), Pescadillo homolog protein (PES1), Cell division cycle protein 2 (CDC2), Glutamine-dependent asparagine synthetase (Ts11), Calnexin (CNX)		
**Cell Paracrine and Intracellular Signalling**	Up	[55]
Solute carrier family 24 member 3 (SLC24A3), Insulin like growth factor binding protein 2 (IGFBP2), Tumor necrosis factor ligand superfamily member 4 (TNFSF4), Ras protein specific guanine nucleotide releasing factor 2 (RASGRF2), Polypeptide N-acetylgalactosaminyltransferase 3 (GALNT3), Gap junction protein alpha 5 (GJA5), WNT1 inducible signalling pathway protein 1 (WISP1), V-Ets erythroblastosis virus E26 oncogene homolog (ERG), V-Yes-1 Yamaguchi sarcoma viral related oncogene homolog (LYN)		
**Cell Adhesion and Migration**	Up	
Transforming growth factor beta 2 (TGF-β2), ST6 N-acetylgalactosaminide alpha-2,6-sialyltransferase 5 (ST6GALNAC5), Collagen type X alpha 1 chain (COL10A1), Synaptopodin (SYNPO), Heparin binding EGF like growth factor (HBEGF)		
**ECM Remodelling**	Up	[59]
Plasminogen activator inhibitor 2 (PAI2), Tissue plasminogen activator (PLAT), Matrix metallopeptidase 1 (MMP1), Dickkopf WNT signalling pathway inhibitor 1 (DKK1), Neuregulin 1 (NRG1), Tissue factor pathway inhibitor 2 (TFP12)		
**Steroid Hormone Metabolism**	Down	[55,57]
Aldo-keto reductase family 1 member C1 (AKR1C1), Aldo-keto reductase family 1 member C2 (AKR1C2), Phosphatidic acid phosphatase type 2B (PPAP2B), Growth hormone regulated TBC protein 1 (GRTP1)		
**Cell Adhesion and Migration**	Down	[55,57]
Slit homolog 3 protein (SLIT3), Osteoblast-specific factor 2 (OSF2), OB-cadherin-1 (CDH11), Cytokine-inducible nuclear protein		
**Tumour Suppressor Genes**	Down	[20,55,58]
Tumour protein P53 (TP53), Cyclin-dependent kinase inhibitor 1A (CDKN1A or P21), Caveolin 1 (CAV1), Phosphatase and tensin homolog (PTEN), Epithelial membrane protein 1 (EMP1)		

**Table 3:** Overview of miRNA dysregulation in BCAFs and its effects on BC progression.

microRNA(s)	Regulation Status in BCAFs	Effects on Breast Tumorigenesis	Ref.
miR-320	Down	Increased ETS2 gene expression resulting in enhanced BC cell invasion and angiogenesis in vitro	[108]
miR-26b, miR-101, miR-141, miR-200b, miR-200c, miR-205, miR-342-3p, let-7g	Down	Enhanced TGF-β signalling in clinically derived BCAFs leading to EMT and BC cell invasion	[99]
miR-31-3p, miR-221-3p, miR-221-5p	Up	Enhanced TGF-β signalling in clinically derived BCAFs leading to EMT and BC cell invasion	[99]
miR-31	Down	Increased SATB2 gene expression resulting in BCAFs invasion and BC cell migration	[39,101]
miR-9	Up	Enhanced BC cell migration, invasion and motility in vitro	[72]
miR-21	Up	Increased Ki-67 protein expression and BC cell proliferation in clinical tumour samples.	[109]
miR-26b	Down	Upregulated IL-6 signalling pathway leadingto enhanced BC cell migration	[110]
miR-221, miR-222	Up	Repressed ER expression which led to lower recurrence-free period and overall survival in BC patients Reduced A20 (aka. TNFAIP3) gene expression subsequently inhibits NF-κB activity resulting in promotion BCAFs and BC cell growth and migration	[111,112]
miR-155	Up	Repressed CD36 in stroma cells leading to the promotion of proliferation, migration and angiogenesis in BC cells	[113]
miR-133b	Up	Promoted BC cell growth through inhibition of apoptosis	[114]
miR-148a	Down	Promoted BC cell migration in vitro	[39]
miR-21, miR-143, miR-378e	Up	Promoted stemness, EMT, anchorage-independent cell growth, and invasive capacity of BC cells in vitro	[71]

Abbreviations: miR, microRNA; BCAF, breast cancer-associated fibroblast; BC, breast cancer; ETS2, ETS proto-oncogene 2, transcription factor; TGF-β, transforming growth factor-beta; EMT, epithelial-mesenchymal transition; SATB2, special AT-rich sequence-binding protein 2; Ki-67, antigen Ki-67; aka., also known as; A20/TNFAIP3, TNF alpha-induced protein 3; CTGF, connective tissue growth factor; NF-κB, nuclear factor kappa-light-chain-enhancer of activated B cells; IL-6, interleukin-6; ER, estrogen receptor; CD36, cluster of differentiation 36.
